# Galanin suppresses visceral afferent responses to noxious mechanical and inflammatory stimuli

**DOI:** 10.14814/phy2.14326

**Published:** 2020-01-20

**Authors:** Toni S. Taylor, Parvesh Konda, Sarah S. John, David C. Bulmer, James R. F. Hockley, Ewan St. John Smith

**Affiliations:** ^1^ Department of Pharmacology University of Cambridge Cambridge UK; ^2^ GSK GSK Medicines Research Centre Stevenage Hertfordshire UK

**Keywords:** colon, galanin, hypersensitivity, inflammation, mechanosensitivity, visceral pain

## Abstract

Galanin is a neuropeptide expressed by sensory neurones innervating the gastrointestinal (GI) tract. Galanin displays inhibitory effects on vagal afferent signaling within the upper GI tract, and the goal of this study was to determine the actions of galanin on colonic spinal afferent function. Specifically, we sought to evaluate the effect of galanin on lumbar splanchnic nerve (LSN) mechanosensitivity to noxious distending pressures and the development of hypersensitivity in the presence of inflammatory stimuli and colitis. Using ex vivo electrophysiological recordings we show that galanin produces a dose‐dependent suppression of colonic LSN responses to mechanical stimuli and prevents the development of hypersensitivity to acutely administered inflammatory mediators. Using galanin receptor (GalR) agonists, we show that GalR1 activation, but not GalR2/3 activation, suppresses mechanosensitivity. The effect of galanin on colonic afferent activity was not observed in tissue from mice with dextran sodium sulfate‐induced colitis. We conclude that galanin has a marked suppressive effect on colonic mechanosensitivity at noxious distending pressures and prevents the acute development of mechanical hypersensitivity to inflammatory mediators, an effect not seen in the inflamed colon. These actions highlight a potential role for galanin in the regulation of mechanical nociception in the bowel and the therapeutic potential of targeting galaninergic signaling to treat visceral hypersensitivity.

## Graphical abstract

Galanin inhibits lumbar splanchnic nerve activity in response to mechanical distension via GalR1 and also abolishes the mechanical hypersensitivity induced by inflammatory mediators. However, galanin loses this inhibitory effect in a model of colitis.

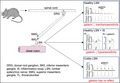

## 1 INTRODUCTION

Visceral hypersensitivity is commonly observed in patients with gastrointestinal diseases, such as irritable bowel syndrome (IBS) and inflammatory bowel disease (IBD), and contributes to the perception of pain in those conditions (Chang et al., 2000; Ritchie, 1973). In the periphery, distal terminals of sensory neurones transduce noxious stimuli to produce nociception and the sensation of pain (Grundy, Erickson, and Brierley 2019; Hockley, Smith, & Bulmer, 2018; St John Smith, 2018). However, a switch from acute to chronic pain and long‐term hypersensitivity can occur due to persistent activation of sensory nerve endings in response to disease‐driven changes in the bowel microenvironment (e.g., the production of inflammatory mediators), coupled with longer‐term changes in the plasticity of the sensory nerve endings and subsequent sensitization of central pain processing pathways. Therefore, factors involved in the development and regulation of visceral hypersensitivity represent potential targets for therapeutic intervention. Early phase pharmacological treatment with antibiotics, corticosteroids, or immunomodulators has been shown to reduce inflammation and in some individuals visceral pain. However, there is still a large subset of patients that still experience discomfort and pain suggesting that visceral pain is discontinuous with the disease activity (Bernstein, 2014). The distal colon receives dual sensory innervation from afferent fibers running within the lumbar splanchnic nerve (LSN) and the pelvic nerve (PN) (Deiteren et al., 2015). Both nerves have been implicated in the processing of nociception from the colorectum, with the PN being identified as the predominant pathway for the signaling of pain in response to rectal distension in humans, and the LSN being identified as the predominant pathway for the signaling of pain in response to distension of the sigmoid and descending colon (Brierley, Jones, Gebhart, & Blackshaw, 2004; Hughes, Brierley, & Blackshaw, 2009; Ray & Neill, 1947). Therefore, the modulation of LSN and PN signaling from respective colonic and rectal regions have important implications in the treatment of chronic visceral pain in GI diseases, such as IBS and IBD (Brierley et al., 2004; Siri et al., 2019).

One method by which colorectal sensory nerves can be modulated is through local release of peptides, calcitonin gene‐related peptide (CGRP) being a widely used marker for such peptidergic sensory nerves, or from nonneuronal cells within the GI tract (Lakhan & Kirchgessner, 2010). We have shown through single‐cell RNA sequencing that the peptide galanin is highly expressed in colonic sensory neurones that also express Trpv1, that is, putative nociceptors and GDNF family receptor alpha‐3 (Gfrα3), that is, high‐threshold stretch‐sensitive afferents, suggesting a potential role in the regulation of colonic nociception (Hockley et al., 2019). Galanin acts upon three receptors, GalR1, GalR2, and GalR3 (Branchek, Smith, Gerald, & Walker, 2000; Branchek, Smith, & Walker, 1998), and single‐cell RNA sequencing demonstrates that GalR1 is the most highly expressed in colonic sensory neurones and that its expression, along with that of GalR2 receptors, is largely restricted to Trpv1‐positive thoracolumbar DRG arising from the LSN, consistent with the hypothesis of a paracrine role for galanin in the regulation of sensory signaling from the bowel (Hockley et al., 2019). Furthermore, GalR1 is also expressed in a population of colonic sensory neurones expressing mechanosensitive ion channel Piezo2, suggesting galanin could modulate LSN activity over a range of pressures. When activated by galanin, GalR1 predominantly signals through G_i_, suggesting that the effects of galanin on colonic afferent signaling would be inhibitory (Branchek et al., 2000). Consistent with this hypothesis, in the upper GI tract of mice and ferrets, galanin suppresses vagal afferent signaling through the activation of GalR1 receptors. Galanin has also been shown to have a stimulatory effect on vagal afferents through the activation of its G_q_‐coupled GalR2 receptor, with GalR3 receptors having no clear functional contribution despite its expression in the relevant ganglia (Page, Slattery, Brierley, Jacoby, & Blackshaw, 2007; Page et al., 2005).

The role of galaninergic signaling in the colorectum is currently unclear and in this study we set out to determine the effect of galanin upon LSN mechanosensitivity due to the high expression of both galanin and its receptors in the relevant thoracolumbar sensory ganglia. Moreover, because single‐cell RNA‐sequencing data demonstrate coexpression of GalR1 with receptors for certain inflammatory mediators (e.g., bradykinin and 5‐hydroxytryptamine) and ion channels such as Trpv1 that are modulated by inflammatory mediators (Hockley et al., 2019), we wanted to determine if galanin could suppress mechanical hypersensitivity induced by inflammatory mediators and extend this to an in vivo model of acute colitis.

We find that galanin is expressed by putative nociceptors originating from the LSN and that it inhibits LSN mechanosensitivity via GalR1. Inflammatory mediator‐induced mechanical hypersensitivity is also abolished by galanin, but suppression of mechanical hypersensitivity is lost in LSN obtained from mice with colitis.

## 2 MATERIALS AND METHODS

### 2.1 Animals and ethical approval

C57BL/6J mice were used for all experiments. All protocols were performed in accordance with the UK Animals (Scientific Procedures) Act 1986 Amendment Regulations 2012 following ethical review by the University of Cambridge Animal Welfare and Ethical Review Body and under UK Home Office Project Licences 70/7705 and P7EBFC1B1. Mice were conventionally housed in groups of 4–5 of the same gender with nesting material and a red plastic shelter; the holding room was temperature controlled (21°C), and mice were on a normal 12 hr/light dark cycle with food and water available ad libitum.

### 2.2 Ex vivo mouse LSN preparation and recording

Adult (8–18 weeks) C57BL/6J male and female mice (Envigo) were humanely killed by cervical dislocation and exsanguination, and the distal colon (from the splenic flexure to rectum) with associated LSN was removed. Colonic content was flushed with Krebs buffer and the colon tied to either end of a cannula, and perfused luminally (200 μl/min) and serosally (7 ml/min) with carboxygenated (95% O_2_, 5% CO_2_) Krebs buffer, in mM: 124 NaCl, 4.8 KCl, 1.3 NaH_2_PO_4_, 2.5 CaCl_2_, 1.2 MgSO_4_·7H_2_O, 11.1 glucose, and 25 NaHCO_3_; supplemented with indomethacin (3 μM, to suppress prostanoid synthesis), and nifedipine (10 μM) and atropine (10 μM) to block smooth muscle contraction as previously described (Hockley et al., 2014). The bath was maintained at 32°C–34°C. The inferior and superior mesenteric ganglia were identified at the point of the iliac bifurcation and suction electrode recordings of multiunit activity were made from neurovascular bundles isolated central to the inferior mesenteric ganglia. Signals were amplified at a gain of 5 K, band pass filtered (100–1,300 Hz), digitally filtered for 50 Hz noise (Humbug, Quest Scientific, Canada), and data acquired at 20 kHz (micro1401; Cambridge Electronic Design, UK). Ongoing nerve discharge was quantified from spikes passing a threshold level set at twice the background noise (typically ~100 μV). All signals were displayed on a PC using Spike 2 software. The baseline pressure was set up at 2–3 mmHg and recordings were maintained for approximately 30 min before initiating the experimental protocols.

Mechanosensitivity was evaluated using ramp and phasic distension protocols. For the ramp distension protocol the luminal outflow cannula was blocked and the subsequent increase in pressure was observed until the desired maximum of 80 mmHg was reached (~2.5 min), at which point the luminal outflow was re‐opened. For the phasic distension protocol, a rapid increase in the intraluminal pressure from 0 to 80 mmHg was achieved by switching the luminal outflow to a water column of sufficient height.

For drug treatments, two protocols were used. In one protocol three phasic distensions were performed to obtain a stable response to distension, and then galaninor vehicle (or the GalR1/2 agonists M617 and spexin, respectively, Tocris) was serosally superfused (20 ml volume) between the third and fourth phasic distension. In the second protocol the effect of galanin (or vehicle) was examined on ramp and phasic distension (an initial ramp distension followed by three phasic distensions) in nonsensitized preparations (continual luminal perfusion with Krebs buffer) or sensitized preparations. Sensitization was achieved by intraluminal perfusion of an inflammatory soup that has previously been shown to increase resting afferent activity (Su & Gebhart 1998:10 μM histamine, 10 μM prostaglandin E_2_, 10 μM 5HT, 1 μM bradykinin, and 1 mM ATP) for 20 min prior to and during subsequent ramp and phasic distensions.

### 2.3 Analysis of electrophysiological recordings

Phasic distension of the colon by 80 mmHg leads to an increase in LSN activity, which consists of an initial peak response that reduces in magnitude to a sustained increase in nerve discharge for the remainder of the distension period. Nerve discharge returning to baseline levels following cessation of the phasic distension. Peak changes and time profiles of LSN activity were determined by subtracting baseline firing (average over 60 s before distension) from increases in LSN activity following distension. During phasic distensions, peak firing was defined as the maximal firing rate observed during distensions, occurring within the first 15 s, and sustained firing frequency was defined as the activity seen during the subsequent 45 s of the phasic distension. Drug effect was determined by comparing an average of the first three distensions to the response of the third postdrug distension. By contrast, ramp distension leads to a slow and steady increase in activity until the end of the distension. Pressure profiles of LSN activity were determined by subtracting baseline firing from peak LSN activity measured every 5 mmHg.

### 2.4 Retrograde labeling of colonic sensory neurones

Retrograde labeling of colonic sensory neurones was conducted as previously described (Hockley et al., 2019). In brief, C57BL/6J mice were anesthetized with isoflurane (4% induction and 1%–2% maintenance) then a midline laparotomy (~1.5 cm incision) performed to reveal the distal colon. Five injections of 0.2‐μL Fast Blue (2% in saline, Polysciences GmbH, Germany) were made into the wall of the distal colon using a glass needle at a rate of 0.4 μL/min using a microinfusion pump (Harvard Apparatus). After the abdominal cavity was flushed with saline to remove any excess Fast Blue dye, the muscle and skin layers were sutured and secured using 4–6 Michel clips. Postoperative care and analgesia (buprenorphine 0.05–0.1 mg/kg) were provided and a glucose‐enriched soft diet provided, with regular checks of body weight. After 3–5 days, the animals were killed using sodium pentobarbital (200 mg/kg i.p.) and transcardially perfused with phosphate‐buffered saline (PBS) followed by paraformaldehyde (4% in PBS; pH 7.4). Dorsal root ganglia (DRG; T13—L1) were removed and further fixed in 4% paraformaldehyde for 30 min at 4°C before cryoprotection in 30% sucrose overnight at 4°C. The tissue was then embedded in Shandon M‐1 Embedding Matrix (Thermo Fisher Scientific), snap frozen in liquid nitrogen, and stored at −80°C until needed. Cryostat (Leica, CM3000; Nussloch) sections (12 μm) were collated across 10 slides (Superfrost Plus, Thermo Fisher Scientific) for each DRG.

### 2.5 Immunohistochemistry

DRG sections were washed with PBS (twice for 2 min) and then blocked using antibody diluent (10% donkey serum, 5% bovine serum albumin, and 0.2% Triton X‐100 in 0.1 M PBS) for 1 hr. This was followed by overnight incubation at 4°C with the appropriate primary antibodies (Table 1, we thank Prof. Theodorsson, Linköping University, for the kind gift of the anti‐galanin antibody Theodorsson and Rugarn 2000). The sections were then washed three times for 5 min with PBS and then incubated for 2 hr at room temperature with the appropriate fluorophore‐conjugated secondary antibodies (Table 2). No labeling was observed in control experiments where the primary antibody was excluded or in the presence of galanin as a blocking peptide for the anti‐galanin antibody.

**Table 1:** List of primary antibodies

Primary antibody	Conc.	Company	Catalogue #	RRID	References
Rabbit anti‐galanin	1:1,000	Theodorrson Lab	Kind gift	–	Sternini et al., 2004)
Guinea pig anti‐TRPV1	1:1,000	Alomone Labs	AGP‐118	AB_2721813	Ritchie, 1973)
Goat anti‐Gfrα3	1:300	R&D Systems	AF2645	AB_2110295	Albers et al., 2014)
Goat anti‐CGRP	1:500	Abcam	AB36001	AB_725807	Branchek et al., 1998)

**Table 2:** List of secondary antibodies

Secondary antibody	Conc.	Company	Catalogue #	RRID
Donkey anti‐rabbit IgG‐Alexafluor‐488	1:1,000	Invitrogen	A‐21206	AB_2535792
Donkey anti‐rabbit IgG‐Alexafluor‐568	1:1,000	Invitrogen	A10042	AB_2534017
Donkey anti‐goat IgG‐Alexafluor‐568	1:1,000	Invitrogen	A‐11057	AB_2534104
Donkey anti‐guinea pig IgG‐Alexafluor‐488	1:1,000	Jackson Immuno Research	706‐165‐148	AB_2340460

### 2.6 Imaging and quantification

Sections were imaged using an Olympus microscope (BX51) with Qicam camera and the relative intensities of DRG neurones after immunostaining were measured (ImageJ 1.51n analysis software, NIH, USA). The mean background intensity was subtracted to control for variability in illumination between images. Percentages of relative intensities were determined by comparison with least intensely (0%) and the most intensely (100%) labeled cells for each section. Relative intensities are calculated by subtracting the relative intensity of the darkest neuronal profile (a) from the relative intensity of the cell of interest (b) and comparing this to the relative intensity of the brightest neuronal profile (c) with the relative intensity of the darkest neuronal profile subtracted: relative cell intensity = (b – a)/(c – a) (Fang et al., 2002); cells with intensity values greater than the mean intensity of the darkest neuronal profiles from all the sections plus five times its standard deviation (*SD*) were considered positively labeled.

### 2.7 Dextran sulfate sodium (DSS) model of induced colitis

C57BL/6J mice of either sex (8–12 weeks old) were weighed 2 days prior to the procedure; weight and stool content/consistency were then monitored daily throughout the treatment. Drinking water supplemented with 3% DSS (40,000 MW, Alfa Aesar) was administered with control mice receiving the same drinking water without DSS. The mice received DSS‐treated water for 5 days after which it was replaced with normal drinking water for a further 2 days. The experimenter was blinded, and solutions labeled A and B being unblinded after results were fully analyzed. Oral administration of DSS leads to weight loss, diarrhea, and blood in the stool, which was scored to produce a disease activity index (DAI) (Table 3) (Manicassamy & Manoharan, 2014). On day 7, mice were killed, and relevant tissue samples, Wallace macroscopic score (Table 4; Fang et al., 2002), and measurements were obtained. An approximate 1‐cm section of colon was removed about 4 cm from the anus and fixed in 4% paraformaldehyde for 4 hr and cryoprotected in 30% sucrose overnight. The tissue was then embedded in O.C.T mounting media (VWR Q‐path Chemicals), snap frozen in liquid nitrogen, and stored at −80°C until needed.

**Table 3:** Disease activity index (DAI)

Score	Weight loss	Stool consistency	Blood in stool
0	None	Normal	Normal
1	1%–5%		
2	5%–10%	Very Soft	Slight bleeding
3	10%–15%		
4	≥15%	Watery diarrhea	Gross bleeding

Note

Scoring for all three criteria produces a maximal score of 12.

**Table 4:** Wallace macroscopic scoring

Score	Criteria
0	No damage
1	Hyperemia, no ulcers
2	Hyperemia and thickening of bowel wall. No ulcers
3	One ulcer without thickening of the bowel wall
4	Two or more sites of ulceration and inflammation
5	Two or more major sites of ulceration/inflammation, or one site of ulceration/inflammation extending ≥1 cm
6–10	If damage covered ≥2 cm along the colon, the score was increased by 1 for each additional centimeter

### 2.8 Histology: H&E with alcian blue staining

Cryostat sections of colon (20 μm) were collected and stored at −20°C until needed. Slides were washed in tap water for 2 min before staining for 5 min with hematoxylin (1:2 dilution with tap water; Sigma), washing in tap water for 3 min followed by 0.3% HCl in ethanol for 30 s, and then immediately washing in tap water for a further 2 min before incubating in tap water until the tissue developed a deep blue appearance. Slides were then stained with alcian blue (1% W/V in 3% acetic acid; Polysciences Inc) for 10 min and washed in tap water for 2 min before being immersed in 100% ethanol for 30 s. Slides were then stained with eosin (Acros Organics) for 90 s, washed in tap water for 1 min, and then dehydrated in 100% ethanol for 30 s followed by 70% ethanol for 30 s. Slides were then cleared using Histoclear (National Diagnostics) for 30 s before mounting coverslips with Mowiol mounting media. Imaging was carried out using a NanoZoomer S60 Digital slide scanner. A histopathological score was made based on presence of inflammatory cells, crypt damage, and ulceration, visualized using H&E staining, and alcian blue to stain the goblet cells in the crypts (Table 5).

**Table 5:** Histopathological assessment of colon sections

Score	Inflammation	Crypt damage	Ulceration
0	None	Crypts intact	None
1	Increased number of granulocytes in lamina propria	Loss of basal one third of crypt	1–2 foci of ulceration
2	Confluence of inflammatory cells extending to submucosa	Loss of basal two thirds of crypt	3–4 foci of ulceration
3	Transmural extent of infiltrate	Entire crypt loss	Confluent or extensive erosion
4	–	Change in epithelial surface with erosion	–
5	–	Confluent erosion	–

Note

Scoring for all three criteria produces a maximum score of 11.

### 2.9 Biotinylated hyaluronan binding protein (HABP) staining

Twenty‐ micrometer colon sections were cut using a cryostat and mounted on glass slides, which were washed twice with PBS‐Tween before being blocked with an antibody diluent solution (0.2% Triton X‐100 and 5% bovine serum albumin in PBS) for 1 hr at room temperature. Slides were incubated in biotinylated hyaluronan binding protein (Amsbio, 1:200) at 4°C overnight and then washed three times in PBS‐Tween and incubated for 2 hr at room temperature with Alexafluor 488 conjugated streptavidin (Invitrogen, 1:1,000). Slides are then washed three more times with PBS‐Tween before being mounted and imaged using an Olympus BX51 microscope and QImaging camera.

### 2.10 Statistics

All statistical analyses were performed using GraphPad Prism 6. IC_50_ values were derived by a sigmoidal dose–response (variable slope) curve using GraphPad Prism software. For ramp distensions, a repeated measures two‐way ANOVA with Sidak's post hoc test was used. Both basal firing and phasic distension data were analyzed using a repeated measures one‐way ANOVA with Tukey's post hoc test. For the validation of the DSS model, DSS data were compared to control data using Student's *t* test. Statistical significance was set at *p* < .05. Data are presented as mean ± *SD*, *N* = number of animals, and *n* = number of cells.

## 3 RESULTS

### 3.1 Galanin suppresses mechanically evoked LSN firing

**Figure 1:**
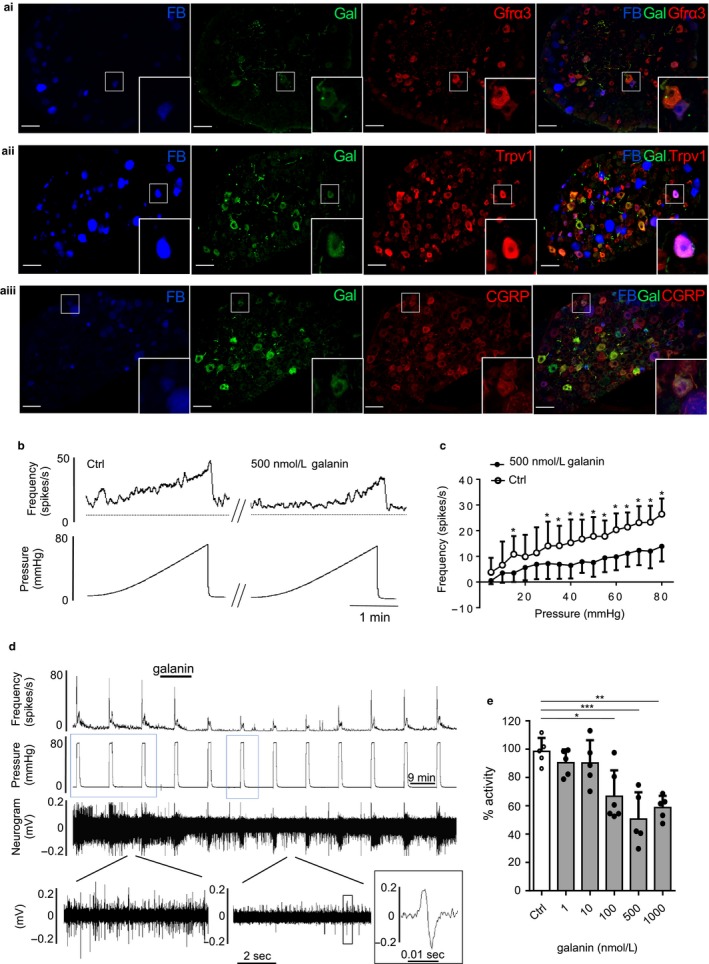
Galanin is expressed in colonic sensory neurones and inhibits LSN mechanosensitivity. (a) Galanin is expressed in TL colonic sensory afferents labeled with fast blue (FB) and is coexpressed with the nociceptive markers Gfrɑ3 (i) and Trpv1 (ii), and the peptidergic marker CGRP (iii); scale bar 50 µm. (b) Example raw trace of ramp distension and response showing inhibition of activity by galanin. (c) Galanin inhibits the LSN response to ramp distension over a wide range of pressure. Firing frequency calculated by subtraction average baseline firing 1 min before distension (two‐way repeated‐measures ANOVA with Sidak's post hoc test, *N* = 6). (d) Example raw trace and frequency histogram of the LSN response to repeated phasic intraluminal distension (0 to 80 mmHg), showing reversible inhibition by galanin. Below, expanded sections of basal activity before (left) and after (right) galanin application and a single action potential (black box). Blue boxes indicating distensions used for analysis. (e) Galanin dose dependently inhibited the peak response to phasic distension (one‐way ANOVA with Tukey's test, *N* = 6 for 100 nM and *N* = 5 for all other groups). *p < .05, **p < .01, ***p < .001

Single‐cell RNA sequencing of colonic sensory neurones suggests that galanin is expressed in putative nociceptors (Hockley et al., 2019) and we confirmed this through immunohistochemistry on TL DRG from mice in which the distal colon had been injected with the retrograde tracer fast blue. Galanin labeled 22.7 ± 8.3% of colonic sensory neurones (*N* = 3, *n* = 466) and 4.5 ± 2.5% of all TL sensory neurones (*N* = 3, *n* = 1,516). When examining coexpression with Gfrα3, the expression of which correlates with Trpv1, a marker for high‐threshold stretch‐sensitive colorectal afferents (Malin, Christianson, Bielefeldt, & Davis, 2009), we observed marked colocalization between Gfrα3 and galanin in colonic sensory neurones: Gfrα3 staining was present in 90.6 ± 1.2% of galanin‐positive TL colonic sensory neurones (*N* = 3, *n* = 71, Figure 1a, left panel) compared with overall Gfrα3 expression of 43.3 ± 7.5% in TL colonic sensory neurones (*N* = 3, *n* = 407). Similarly, the nociceptive marker Trpv1 was also highly enriched in galanin‐positive TL colonic sensory neurones (97 ± 2.5% *N* = 3, *n* = 48, Figure 1a, middle panel) compared with overall expression of 48.7 ± 3.3% (*N* = 3, *n* = 380) of TL colonic sensory neurones. Lastly, galanin was highly coexpressed with the peptidergic neuronal marker CGRP in colonic sensory neurones: 86.5 ± 6.7% galanin‐positive TL colonic sensory neurones (*N* = 3, *n* = 47, Figure 1a, right panel). These results align with RNA‐sequencing data and confirm that galanin is highly expressed in nociceptive colonic sensory neurones.

To determine the effect of galanin on LSN mechanosensitivity, we measured the effects of 500 nM galanin on the response to a ramp distension of the colon (0–80 mmHg) and observed a robust inhibition of spike frequency across the pressure range used, an effect that was most pronounced at 80 mmHg (Figure 1b and c). We therefore used a rapid phasic distension of the colon to noxious pressure (80 mmHg) to investigate the dose‐dependent effect of galanin upon LSN mechanosensitivity. Galanin inhibited LSN firing in a dose‐dependent manner, with the response to galanin being most marked on the third phasic distension after galanin infusion (subsequent values are therefore taken from this third distension, Figure 1d). The maximal suppression of the peak afferent response to phasic colonic distension was observed following the application of 500 nM galanin which produced an approximate 50% reduction in afferent mechanosensitivity (53.3 ± 12.8 spikes/s vs. 25.2 ± 5.2 spikes/s, *N* = 5, *p* < .0008, one‐way ANOVA with Tukey's test) and the IC_50_ calculated from the data was 65.7 nM (Figure 1e). A similar suppression by 500 nM galanin was observed of the sustained firing that occurred during the last 45 s of the distension (24.5 ± 8.6 spikes/s vs. 9.3 ± 4.3 spikes/s, *N* = 5, *p* = .013, unpaired *t* test).

### 3.2 Galanin suppresses noxious mechanically evoked neuronal excitation via GalR1

**Figure 2:**
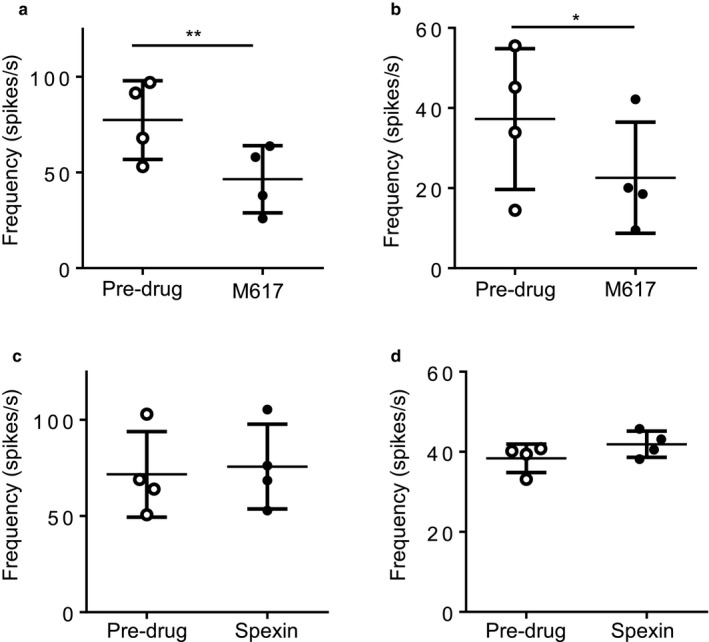
GalR1 activation suppresses LSN responses to phasic distension. (a) The GalR1 agonist M671 significantly attenuates both LSN peak (*p* = .0015, *N* = 4, paired *t* test) and sustained (b; *p* = .0382, *N* = 4, paired *t* test) firing in response to phasic distension of the colon to 80 mmHg. (c) The GalR2 agonist Spexin does not significantly attenuate or excite the peak (c; *p* = .245, *N* = 4, paired *t* test) or sustained (d; *p* = .218, *N* = 4, paired *t* test) response to phasic distension of the colon to 80 mmHg. Firing frequency calculated by subtraction average baseline firing 1 min before distension. *p < .05, **p < .01

Having determined that galanin suppressed colonic afferent mechanosensitivity we next investigated the effect of GalR1 and GalR2 agonists on LSN activity. Using the same protocol as for galanin, the selective GalR1 agonist M617 (500 nM) elicited a reduction in LSN mechanosensitivity comparable in magnitude to galanin at 500 nM, such that the peak LSN response to mechanical distension was suppressed from 77.4 ± 10.3 spikes/s versus 46.5 ± 8.8 spikes/s (Figure 2a; *p* = .0015, *N* = 4, paired *t* test) and the sustained response was also suppressed from 37.3 ± 8.8 spikes/s versus 22.6 ± 6.9 spikes/s (Figure 2b; *p* = .038, *N* = 4, paired *t* test). The effect of 500‐nM M617 was similar to that of 500 nM galanin: M671 attenuated peak firing frequency by 41.4 ± 7.5% and 500 nM galanin suppressed peak firing frequency by 49.3 ± 16.9% (M617 *N* = 4, galanin *N* = 5). By contrast, 1 µM of the endogenous GalR2 agonist spexin, which shows no binding to GalR1 (Kim et al., 2014), had no effect on LSN colonic afferent mechanosensitivity in response to phasic distension of the colon (Figure 2c and d). These results show that galanin acts via GalR1 to suppress LSN activity in response to distension of the colon.

### 3.3 Galanin suppresses mechanical hypersensitivity induced by inflammatory mediators

**Figure 3:**
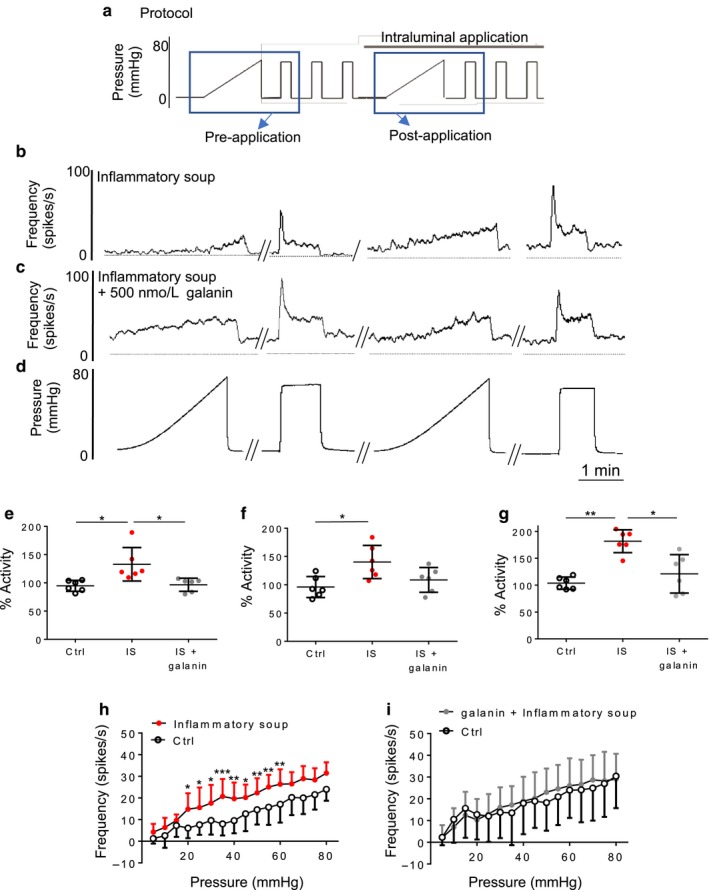
Galanin suppresses the effects of IS on LSN mechanosensitivity. (a) Schematic of pressure protocol used in the multiunit recordings. Ramp and phasic distensions demonstrated below indicated with blue box, intraluminal application of drug indicated by black bar above the protocol. Example raw trace of ramp and phasic distension before and after the intraluminal application of inflammatory soup (b), a combination of Inflammatory soup and galanin (500 nM, c), and sample pressure distensions (d). Changes in peak (e), sustained (f), and basal (g) firing after intraluminal application of either inflammatory soup or a combination. Significant differences between groups tested by one‐way ANOVA with Tukey's test, *p < .05, **p < .01 (*N* = 6). Response profiles to ramp distension before and during intraluminal application of inflammatory soup (h) or a combination of IS and galanin (i, two‐way repeated‐measures ANOVA with Sidak's post hoc test, *N* = 6). Firing frequency calculated by subtraction average baseline firing 1 min before distension. Significance indicated by ^#^ *p* < .05, *p < .01, ~ *p* < .001

We next investigated the effect of galanin on the sensitization of the LSN afferent response to colonic distension by intraluminal perfusion with an inflammatory soup (IS: ATP, histamine, PGE_2_, bradykinin, and serotonin) (Hockley et al., 2014; Su & Gebhart, 1998). The IS was intraluminally perfused following the third phasic distension (either alone or with 500 nM of galanin, Figure 3a–d). As expected, IS significantly increased the peak firing frequency in response to phasic distension (31.2 ± 28.1%, *p* < .05, *N* = 6, one‐way ANOVA with Tukey's test, Figure 3e), whereas coapplication of IS and galanin did not produce a significant change in peak afferent firing compared with control (Figure 3e). A similar effect was observed with regard to the sustained firing frequency, such that IS application significantly increased sustained firing frequency (40.3 ± 26.7%, *p* < .05, *N* = 6, one‐way ANOVA with Tukey's test), which was not observed when IS was combined with 500 nM galanin (Figure 3f). Lastly, we also observed that the basal LSN activity increased by 81.6 ± 19.3% following IS application (*p* < .01, *N* = 6, one‐way ANOVA with Tukey's test), but that combination of IS and galanin did not significantly change the basal firing from the control group, suggesting that the effects of galanin and IS cancel each other out when coadministered (Figure 3e).

To further investigate LSN hypersensitivity caused by IS application and how this is affected by galanin, we used a ramp distention (0–80 mmHg over ~2.5 min). As expected, IS significantly increased activity across a broad range of pressures (Figure 3h, *N* = 6), and again the combination of IS and galanin resulted in a pressure‐induced activity relationship that was not significantly different from control (Figure 3i, *N* = 6). These results demonstrate that galanin suppresses inflammatory mediator‐induced mechanical hypersensitivity of LSN afferents in the colon.

### 3.4 Galanin does not suppress DSS‐induced mechanical hypersensitivity

**Figure 4:**
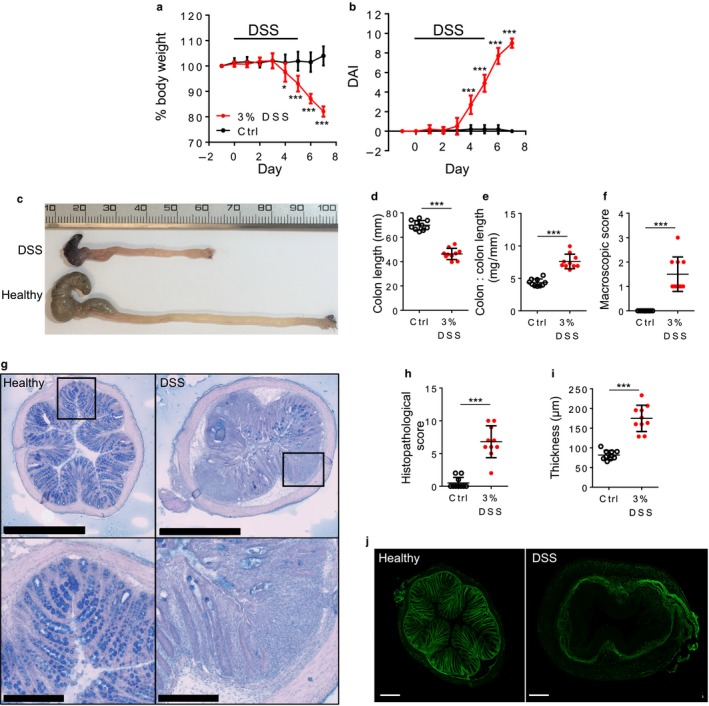
DSS induces weight loss and macroscopic changes to colon histology. (a) Body weight of DSS‐treated mice was significantly reduced compared to untreated controls (*p < .05, ***p < .001, *N* = 10, two‐way repeated‐measures ANOVA with Sidak's post hoc). (b) DAI significantly increases in colitis‐induced mice (***p < .001, *N* = 10, two‐way repeated‐measures ANOVA with Sidak's post hoc test). DAI = disease activity index; assessment of inflammation by clinical parameters. (c) Photograph of healthy and colitis‐induced colons from cecum (left side) to anus (right side). (d) Colon length is significantly reduced in DSS mice (*p* < .001, *N* = 10, paired *t* test). (e) Colon weight to length ratio significantly increased in DSS mice (*p* < .001, *N* = 10, paired *t* test). (f) Macroscopic score is based on visual assessment of ulceration and hyperemia of the colon (*p* < .001, *N* = 10, paired *t* test). (g) H&E with alcian blue staining of colonic tissue. In DSS mice, there was active inflammation, and crypt or surface epithelial damage compared to untreated controls. Areas defined by black boxes are magnified in the lower images; scale bar for top images is 1 mm and for bottom images 250 μm. (h) Histology score significantly increases in DSS mice (*p* < .001, *N* = 10, paired *t* test) and the colonic muscle layer becomes significantly thicker in DSS mice (i, *p* < .001, *N* = 10, paired *t* test). (j) Changes in hyaluronic acid binding protein (HABP; green) arrangement and distribution in colitis‐induced and healthy colons

Using the DSS model of colitis, we observed that DSS‐treated mice showed significant weight loss from day 4 onwards compared with untreated mice (e.g., on day 7, 82.1 ± 1.9% vs. 103.9 ± 3.6% of starting weight, *N* = 10, *p* < .0001, unpaired *t* test) (Figure 4a), and also a significant increase in disease activity index (DAI, Figure 4b). Following dissection, colon length was observed to be significantly decreased (Figure 4c and d) and the colon wet weight to length ratio also significantly increased in DSS mice compared with the control group (Figure 4e). We observed both significant macroscopic and histological damage (Figure 4f–h) in colon sections from DSS mice compared with those from healthy mice, as well as significant thickening of the muscular layer of the colon in the DSS group (Figure 4i) as others have observed (Marrero, Matkowskyj, Yung, Hecht, & Benya, 2000; Sánchez‐Fidalgo, 2012). Lastly, the extracellular matrix polysaccharide hyaluronan (HA) was largely absent from the colon epithelium of DSS‐treated mice (Figure 4j).

**Figure 5:**
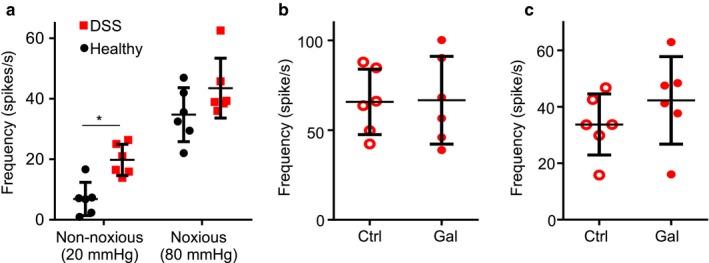
Galanin does not suppress DSS‐induced LSN hypersensitivity. (a) DSS increases the response to nonnoxious (20 mmHg) but not noxious (80 mmHg) pressures using a ramp distension protocol; Student's *t* test between groups *p < .05. Galanin of 500 nM does not inhibit peak (b) or sustained (c) responses to phasic distension of the colon to 80 mmHg in colons isolated from DSS‐treated mice (paired *t* test, *N* = 6). Firing frequency calculated by subtraction average baseline firing 1 min before distension

Using the DSS model, we investigated if the suppression of LSN activity by galanin in healthy mice (Figure 1) was maintained in LSN from mice undergoing DSS‐induced colonic inflammation. Using a ramp distention, we observed that the LSN isolated from DSS‐treated mice produced a greater response at nonnociceptive pressure (20 mmHg, 6.9 ± 5 spikes/s vs. 19.8 ± 4.7 spikes/s, *p* = .0184, *N* = 6, *t* test), but not at nociceptive pressure compared to the LSN from healthy mice (34.7 ± 8.1 spikes/s vs. 39.7 ± 3.2 spikes/s, *p* = .137, *N* = 6, Student's *t* test; 80 mmHg, Figure 5a). In addition, the basal firing of LSN from DSS‐treated mice was significantly greater than that of healthy mice (3.9 ± 1.4 spikes/s vs. 28.4 ± 13.4 spikes/s, *p* = .012, *N* = 6, Student's *t* test). Together with the ramp distension response data, these results suggest that DSS induced a state of visceral hypersensitivity at the level of the primary afferent. Whereas galanin was observed to significantly suppress the peak and sustained LSN responses induced by a phasic distension to 80 mmHg of the colon in healthy mice (Figure 1), no such suppressive effect was observed when measuring LSN activity in nerves isolated from DSS mice: peak firing frequency (65.7 ± 16.6 spikes/s vs. 66.6 ± 22.3 spikes/s, *p* = .81, *N* = 6, paired *t* test; Figure 5b) and sustained firing frequency (34.1 ± 9.9 spikes/s vs. 41.9 ± 14 spikes/s, *p* = .54, *N* = 6, paired *t* test; Figure 5c) induced by a phasic distension to 80 mmHg were affected by administration of galanin. These results indicate that the suppressive action of galanin is lost in tissue isolated from mice with acute colitis.

## 4 DISCUSSION

Our data demonstrate that galanin suppresses LSN responses to noxious mechanical stimuli and the sensitization of colonic mechanosensitivity by acute application of inflammatory mediators. This suppressive effect of galanin was not seen in tissue isolated from mice with colitis. Our data further suggest that GalR1 mediates the suppressive effect of galanin. These data fit alongside galanin's reported function in the upper GI tract (Page et al., 2005) and parallels with the function of galanin in somatic sensory innervation (Flatters, Fox, & Dickenson, 2003; Heppelmann, Just, & Pawlak, 2000).

Galanin is expressed by multiple cell types in the distal colon including enterochromaffin cells and fibroblasts (Schäfermeyer et al., 2004; Yamamoto, Iguchi, Unno, Kaji, & Hoshino, 2014), and, as we show here, a subset of colonic sensory neurones also express galanin. Galanin has been implicated as a modulator of numerous activities in the GI tract including regulation of neurotransmitter release, motility, and secretion (Benya, Marrero, Ostrovskiy, Koutsouris, & Hecht, 1999; Sternini et al., 2004). With regard to the transmission of sensory information from the GI tract, galanin has been shown to modulate mechanosensitivity of gastrooesophageal vagal afferents with predominantly suppressive actions on individual fibers via GalR1 (Page et al., 2007, 2005). Our data build on this by demonstrating that galanin suppresses LSN mechanosensitivity and likely does so though GalR1 activation on LSN afferents. Another possibility to consider is that the effects of galanin occur via secondary actions resulting from paracrine signals from nonneuronal calls. In inflammation, mediators released from activated enteric mast cells have the potential to sensitize colonic afferents to become hyperexcitability to stimuli such as colonic distension (Wang et al., 2014; Wood, 2011). Galanin is known to suppress paracrine signal transmission in the gut (Liu, 2003; Tamura, Palmer, & Wood, 1987) and with GalR expression being found in immune cells in the colon could suggest that galanin release suppresses neurotransmission from afferents to mast cells and suppresses release of sensitizing mediators from the mast cells themselves. Mechanical distension of the colon is capable of producing pain in humans and nociceptive behavior in animals (Gebhart, 2004; Ness, Metcalf, & Gebhart, 1990). Consequently, distension is a useful tool when defining noxious responses in afferent preparations and examining hyperexcitability to colonic inflammation. We find from multiunit recordings of LSN activity that galanin suppresses mechanically evoked responses to distension of the distal colon to noxious pressures, and the sensitization of these responses following addition of inflammatory stimuli. This suggests that galanin receptors, specifically in the case of the LSN GalR1, function as integral regulators of neuronal excitability. Multiple populations of sensory afferents innervate the distal colon with differing sensitivities to mechanical stimuli (e.g., stretch, stroke, and von Frey hair probing of their receptive fields) (Brierley et al., 2004) and while we did not seek to characterize these groups more specifically, we did observe suppressive effects of galanin across the full range of distension pressures from physiological through to noxious (i.e., 0–80 mmHg, Figure 1c). When examining the expression of galanin, both colonic sensory neurone RNA sequencing and immunohistochemistry (Figure 1a) demonstrate that galanin is expressed in putative nociceptors in healthy animals (Hockley et al., 2019). With regard to GalR1, colonic sensory neurone RNA sequencing indicates that it is predominantly expressed in neurones expressing nociceptor markers, such as Trpv1 and Gfrα3, as well as in a population of neurones expressing the mechanosensitive ion channel Piezo2, an expression pattern that likely explains the suppressive impact of galanin on LSN activity across a wide range of pressures (Figure 1c). The GalR1 receptor is coupled to G protein‐coupled inwardly rectifying potassium channels (GIRKs) giving rise to hyperpolarization (Smith et al., 1998; Walker et al., 1997), an effect that would account for the suppressive activity of galanin observed in this study. This conclusion is further supported by data demonstrating that suppression of LSN activity was also produced by the GalR1 agonist M617, but not the GalR2 agonist spexin (Figure 2).

As observed in previous studies (Su & Gebhart, 1998), we found that intraluminal application of IS produced robust LSN hyperexcitability to mechanical stimuli (Figure 3). Part of this hyperexcitability likely results from recruitment of ‘silent’ or mechanically insensitive afferents in both the LSN and PN (Feng & Gebhart, 2011), potentially through disinhibition of Piezo2 (Prato et al., 2017). The ability of galanin to reverse the mechanically hypersensitivity induced by the IS correlates with the fact that a variety of inflammatory mediator receptors are present in colonic sensory neurones that also express GalR1 (Hockley et al., 2019). However, by contrast, galanin was not able to reduce the mechanical hypersensitivity present in LSN isolated from mice treated with DSS to induce a state of colitis.

Why is it that galanin counteracts the acute effects of inflammatory mediators on LSN activity, but has no such effect on LSN activity in a mouse model of colitis? It has been observed that galanin receptor expression is altered in certain models of inflammation and hence differential receptor expression could lead to galanin no longer exerting a suppressive effect. For example, following hind‐paw injection of carrageenan in rats, GalR1 mRNA expression in DRG neurones (L4 and L5) decreases (Xu, Shi, Landry, & Hökfelt, 1996) and a similar decrease in GalR1 expression could occur in the DSS model and hence galanin is no longer able to suppress mechanically evoked LSN activity; alternatively, there could be an increase in the expression of excitatory GalR2. The absence of reliable tools to investigate GalR protein levels and the validity of current antibodies being uncertain (Lu & Bartfai, 2009) make quantifying the protein level of GalRs in colonic afferents a significant challenge. However, there are also alternative explanations for the lack of measurable galanin activity. For example, multiple inflammatory mediators are released from inflamed colon tissue (Spiller & Major, 2016), which would act upon a broad range of afferents to induce a variety of transcriptional changes and posttranslational modifications, that is, both GalR1+ve and GalR1‐ve afferents, and thus the mechanical hypersensitivity observed in LSN isolated from DSS‐treated mice is likely at least partially mediated via GalR1‐ve afferents. Therefore, any effect of galanin on the whole‐nerve response may simply be overcome by the overall level of sensitization. A further explanation would be that the coupling of GalR1 is altered in inflammation due to altered expression of G proteins and/or that the signaling of galanin at GalR1 becomes biased toward different pathways. Further studies examining the effects of galanin on LSN hypersensitivity in other models of visceral pain (e.g., colonic instillation of trinitrobenzenesulfonic acid) would also be beneficial to determine if the loss of galanin's suppressive effect is common to animal models, which would lend further insight for potential translation.

In conclusion, we have shown an unreported role for galanin in the modulation of LSN function in the distal colon: galanin suppresses LSN mechanosensitivity and acute mechanical hypersensitivity induced by an IS. Future work should elucidate mechanisms underpinning why galanin is unable to exert any suppression on LSN mechanical hypersensitivity following prolonged (in vivo) inflammation.

## CONFLICT OF INTEREST

The authors declare that they have no competing interests.

## AUTHOR CONTRIBUTIONS

TST, JH, and ESS conceived and designed the research. TST performed the experiments and analyzed the data with assistance from PK and SJ. TST drafted the manuscript and all authors edited, revised, and approved the final version of the manuscript submitted for publication. All authors agree to be accountable for all aspects of the work in ensuring that questions related to the accuracy or integrity of any part of the work are appropriately investigated and resolved. All persons designated as authors qualify for authorship, and all those who qualify for authorship are listed.
