# Supplementary material for: Galanin suppresses visceral afferent responses to noxious mechanical and inflammatory stimuli
Source: Physiol Rep. 2020 Jan 20;8(2):e14326. doi: 10.14814/phy2.14326 (PMC6971316; doi:10.14814/phy2.14326)
Supplement: Supplementary file 1 [file PHY2-8-e14326-s001.pptx]

## Slide 1
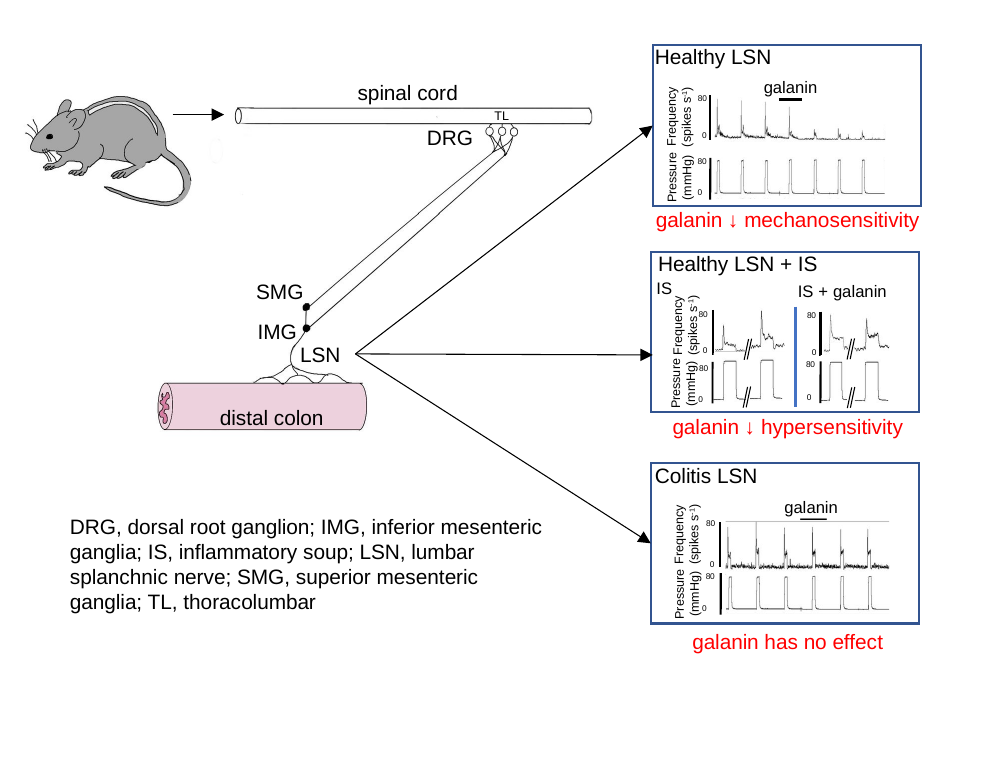

Healthy LSN
galanin
spinal cord
80
Frequency
(spikes s-1)
TL
DRG
0
80
Pressure
(mmHg)
0
galanin ↓ mechanosensitivity
Healthy LSN + IS
IS
SMG
IS + galanin
80
80
Frequency
(spikes s-1)
IMG
LSN
0
0
80
80
Pressure
(mmHg)
0
0
distal colon
galanin ↓ hypersensitivity
Colitis LSN
galanin
DRG, dorsal root ganglion; IMG, inferior mesenteric ganglia; IS, inflammatory soup; LSN, lumbar splanchnic nerve; SMG, superior mesenteric ganglia; TL, thoracolumbar
80
Frequency
(spikes s-1)
0
80
Pressure
(mmHg)
0
galanin has no effect
